# How public health practitioners in the UK are using parental guidance on talking to children about weight: a qualitative study

**DOI:** 10.1136/bmjopen-2025-105371

**Published:** 2026-02-25

**Authors:** Rowan Brockman, Fiona Gillison, Elisabeth B Grey, Russ Jago, Georgie J MacArthur, Callum Gutteridge, Rebecca Langford

**Affiliations:** 1 The National Institute for Health and Care Research Applied Research Collaboration West (NIHR ARC West), Bristol, UK; 2 University of Bristol, Bristol, UK; 3 University of Bath, Bath, UK; 4 Gloucestershire County Council, Gloucester, UK

**Keywords:** parents, obesity, overweight, child, qualitative research, public health

## Abstract

**Abstract:**

**Objective:**

To understand how public health practitioners (PHPs) are using parental guidance on talking to children in their work with parents. In 2021, evidence-based guidance was produced for parents of young children to facilitate these conversations, but it is unclear how this guidance is being promoted to parents or used by PHPs.

**Design:**

Qualitative study, consisting of in-depth, semistructured interviews.

**Setting:**

Local authority, National Health Service or other healthy weight service providers in the UK.

**Participants:**

Participants were PHPs working on children’s healthier lifestyles programmes in the UK as part of the UK’s National Child Measurement Programme (NCMP). Invitations to participate were distributed via the Department of Health and Social Care and regional and national networks.

**Results:**

24 participants were interviewed. Practice varied between organisations with the guidance being used in NCMP letters to parents, in follow-up phone calls with parents and in training NCMP staff and other health or education professionals. Participants valued the evidence-based guidance and its compassionate tone, feeling it gave them and parents, confidence in addressing a sensitive topic. Some felt it was too lengthy for parents with learning disabilities or low literacy levels. Others identified a need for similar guidance for older children. Though helpful, participants acknowledged the guidance was only one small part of a necessary systems-wide approach to promoting healthy weight.

**Conclusions:**

The guidance is a useful tool but needs systematic promotion to increase use and effectiveness. Further work is warranted to develop adapted versions for other populations.

**STRENGTHS AND LIMITATIONS OF THIS STUDY**

- Semistructured interviews offered valuable insights into how practitioners used the guidance to support parents in discussing weight with their children.
- We recruited participants from across England and Scotland, representing a range of roles and organisations.
- We identified key areas for future development, including the need for tailored guidance for adolescent populations.
- Participants reported variation in awareness and uptake of the guidance, meaning that more work is needed to better embed the resource into core National Child Measurement Programme procedures.

## Introduction

 Nearly one in four (23.5%) children starting primary school (aged 4–5 years) in England are living with overweight or obesity. By the end of primary school, this figure rises to over one in three (36.1%).^1^ Children in the most deprived areas are over twice as likely to live with obesity than the most affluent children.^2^ Children living with overweight or obesity are more likely to be bullied,^3^ experience low self-esteem and body dissatisfaction,^4^ and obesity in adulthood,^5^ which increases the risk of chronic disease.^6^

In England, the National Child Measurement Programme (NCMP) annually monitors the prevalence of overweight and obesity in Reception (4–5 years) and Year 6 (10–11 years) children across local authorities (LAs), unless parents/caregivers withdraw their child^1^ (hereafter referred to as parents). In most LAs, measurement teams inform parents if their child is overweight or underweight, often providing links to further support, such as online information or details of local child weight management programmes (also known as healthier lifestyle programmes).^7^

Participating in the NCMP may prompt conversations about weight which parents may find challenging. Parents fear such conversations may damage their child’s self-esteem or contribute to the development of eating disorders.^8 9^ While many parents wish to help their child maintain a healthy weight, research highlights parents’ lack of confidence in communicating positively about weight,^10^ a point reiterated by the public contributors on this project. Consequently, practitioners delivering the NCMP have expressed a need for guidance for parents to assist them with these conversations.^11^

To address this parental need, a team of academics developed the guidance document, ’, first published in 2021 and updated in 2023.^12^ Aimed at parents of children of any weight, it seeks to increase parental confidence in handling weight-related conversations. It was developed drawing on: systematic reviews on parent communication about weight/health^13 14^; interviews with children; key message development^15^; and a modified Delphi process with parents, health professionals and academics. A description of this process is available^16^ and the guidance content is outlined in box 1.

Box 1 **Guidance overview**

**Talking to your child about weight: A guide for parents and caregivers of children aged 4–11 years.^12^**

The aim of this guide is to help parents and caregivers talk with their children about weight in a positive way. It gives tips and advice on what to say and do to help children be healthy and feel good about their bodies.

This guidance is for parents and caregivers of children of all shapes and sizes.

Section 1: Should I talk to mychild about their weight?

Section 2: The whole family counts.

Section 3: Top tips for talking to your child about weight.

Section 4: How to help your child feel good about their body?

Section 5: What if I am struggling with my own weight?

Section 6: What could I say when…?

Section 7: Links to further advice and information.

### Contents page reproduced with permission of the authors

The guidance was developed with the intention that staff involved with NCMP delivery would signpost parents to it. Feasibility research in 2021 with school nurses identified several methods and stages in the NCMP process where the guidance would be useful.^11^ An e-learning course was created for public health practitioners (PHPs) and other staff, to build confidence in using the guidance with parents.^17^ With no funding for broad dissemination, the guidance was included in a toolkit by the British Dietetic Association^18 19^ and hosted on the University of Bath website.^12^ Guidance authors felt this was a pragmatic solution that could increase parents’ trust in the resource, given these were professional and academic organisations.

The guidance was adopted by England’s Department of Health and Social Care (DHSC) and promoted to school nurses and NCMP delivery teams through regular briefings ahead of the 2021/2022 school year. The resource was also promoted to LA staff and school nurses at four regional and national DHSC events between 2021 and 2024, and other network meetings hosted by Obesity UK (2021) and Food Active (2021/2023). A link to the guidance was added to template NCMP letters in 2024 indicating its endorsement, but with no further promotion to NCMP leads and relevant groups for it to be embedded in practice. Consequently, it is unclear how it is being shared with parents or used by child weight management professionals.

This qualitative study had two key aims:

1. To understand if and how the guidance is currently used in child weight management services.
2. To explore barriers and facilitators to its implementation.

## Methods

### Study design

We used a qualitative study design (interviews) with practitioners working in child weight management and NCMP delivery. This method allowed for an in-depth understanding of how the parental guidance was being used in a range of settings and services.

### Participants and recruitment

We recruited interview participants by contacting professionals working in child weight management and NCMP delivery. While the NCMP only runs in England, health practitioners in Wales and Scotland who run similar programmes and had received the guidance were also eligible. We outlined the study and invited them to participate in an interview. Participants could participate regardless of prior or current use of the guidance.

Participants were contacted through multiple channels: DHSC promoted the study in their February and April 2024 updates to NCMP regional leads, and regional leads shared it with local healthy weight networks. At a January 2024 DHSC webinar, 345 stakeholders (LA public health teams, PHPs and school nurses) were invited to participate. The study was also shared through our own networks of those known to be familiar with or using the guidance. Participants contacted RB/FG to arrange an interview. We interviewed all participants who expressed an interest.

#### Data collection

Interviews were conducted online between February and May 2024 by RB, allowing her to develop familiarity and a comprehensive view of the dataset before analysis. RB is a female, postdoctoral researcher with 18 years of experience in qualitative research on a range of health topics, including weight management and physical activity in children. Participants had no prior knowledge or previous relationship with the interviewer. Some participants requested a joint interview with other team members due to shared roles. While there are notable disadvantages to dyadic interviews, such as sensitive disclosures being inhibited due to a colleague’s presence and the relational nature of the data making coding and interpretation more complex, they have distinct advantages resulting from participants having a shared interest in the topic and a pre-existing relationship between them. For example, the presence of two participants allows them to build on or challenge each other’s accounts, creating more layered and nuanced narratives than a single interview might produce. Additionally, dyads can reproduce natural conversation patterns, making the data feel more authentic and reflective of real-world interaction.^20^,^23^ We used a broad topic guide focusing on participants’ awareness of the guidance, if/how it was used, perceived impact on parents, plans for future use and relevance for their local population (see online supplemental file 1). Interviews lasted 23–62 min, were audio-recorded and transcribed verbatim. Data were stored on secure servers. Participants were offered a £25 voucher for their time.

#### Data analysis

Transcripts were anonymised and analysed using codebook thematic analysis.^24^ RB and RL independently reviewed three transcripts, noting potential codes to develop an initial coding framework. Codes were developed deductively from research questions and inductively from participant responses. RB and RL used this framework to independently code two transcripts (using NVivo) revealing consistency in coding and interpretation. RB coded all subsequent transcripts, making additions/modifications where necessary. Analysis was led by RB, with regular discussion with RL, as well as with the wider study team and our public contributors. Codes and subcodes were examined, compared and revised to develop three descriptive themes. The COREQ (Consolidated criteria for Reporting Qualitative research) checklist is provided as online supplemental file 2.

Quotes are labelled P (for interview participant) followed by the participant number.

### Public involvement

We recruited four public contributors who were parents/carers of primary-school aged children (of any weight status) via Obesity Voices and People in Health West of England. Due to project timings, public contributors were not involved in the study design but offered perspectives on the results and contributed to the focus of the discussion.

## Results

### Participants

Participant characteristics are shown in table 1. 24 participants were interviewed across 16 interviews; all but 1 were female. 10 participants were recruited via the survey, with the remaining recruited via regional NCMP leads and study team contacts. 14 were employed by LAs (representing six different LAs); 9 worked for the National Health Service; and 1 was employed by a community interest company.

**Table 1:** Characteristics of interview participants

Participant	Job role	Organisation	Region	Single/joint interview	Use for NCMP guidance
1	Healthy weight practitioner	NHS	Scotland	Single	Training and delivery of NCMP
2	Manager, health services provider	CIC	Yorkshire and Humber	Single	Training and delivery of NCMP
3	Community nurse	NHS	London	Single	Not currently used
4	Public health practitioner	LA	West Midlands	Joint	Training
5	Public health practitioner	LA	West Midlands		Training
6	Public health practitioner	LA	West Midlands		Training
7	Public health practitioner	LA	South West	Joint	Training
8	Public health practitioner	LA	South West		Delivery of NCMP
9	Public health practitioner	NHS	Merseyside	Single	Training
10	Health psychologist	LA	East Midlands	Joint	Training, delivery and sharing resource with stakeholders (eg, education and health professionals)
11	Service development officer	LA	East Midlands		Delivery of NCMP
12	Policy officer	LA	London	Joint	Delivery of NCMP
13	Policy manager	LA	London		Delivery of NCMP
14	School nursing lead	LA	South West	Single	Training and delivery of NCMP
15	Healthy weight team	LA	West Midlands	Joint	Training
16	Healthy weight team	LA	West Midlands		Training
17	Healthy weight team	LA	West Midlands		Training
18	Service manager	LA	West Yorkshire	Single	NCMP delivery
19	Healthy weight specialist	NHS	East Midlands	Single	NCMP training and delivery
20	Public health team lead	LA	London	Single	Training, delivery and sharing resource with stakeholders (eg, education and health professionals)
21	School nurse associate	NHS	South West	Single	Training
22	School nursing manager	NHS	South West	Single	NCMP training and delivery
23	Health and well-being team lead	NHS	West Yorkshire	Joint	NCMP delivery
24	Health and well-being practitioner	NHS	West Yorkshire		NCMP delivery

CIC, Community Interest Company; LA, local authority; NCMP, National Child Measurement Programme; NHS, National Health Service.

### How the guidance is currently used

The guidance was mainly used in NCMP letters, parent phone calls and as a training resource.

### NCMP parent letters

The guidance was often included in NCMP information sent to parents, either in the invite letter (sent to all Reception/Y6 parents) or the child’s results letter (sent out if the child is identified as overweight or underweight). Letters either included a direct link to the guidance or directed parents to the organisation’s website where they could find the link:

[It’s] linked into our NCMP letters, …and we say, ‘If you want to talk to your child about their weight, here is a resource that you can use.’ (P20)

Some participants felt the guidance link should be included in the NCMP invitation letters so all families could access it. However, several NCMP organisations did not mention the guidance in their standard communications with parents. In some cases, there appeared to be an element of professional gatekeeping, with participants suggesting they were selective about which parents it would be appropriate for:

When I know a parent will be able to cope with the length of that document I will send it to them, but… I’m not going to send it to a parent where I know there’s literacy issues or that they will not sit and read a 12 [sic] page document. (P01) (NB: the official document is 20 pages).

### Follow-up phone calls to parents

In some regions, the guidance was used in phone calls with parents whose child had been identified as outside of the healthy weight range. The guidance was either mentioned during the call or sent to parents afterwards. As one participant explained, parents often worry about sharing the NCMP result with their child, making it a suitable time to introduce the guidance:

If a parent’s not sure, should I speak to my child? … We will suggest you do what you think is best for you and your child. However, we have this [guidance], which is brilliant, that you can read through. It’s got lots of ideas if you’re not sure how to start that conversation. (P19).

Another found the guidance useful in starting conversations with parents and in ‘manag[ing] any resistance or frustration as it can be a sensitive topic’. (P16)

### Training and supporting health and education professionals

Participants discussed how the guidance was used to train staff delivering the NCMP. For example, although aimed at parents, one LA used the guidance in a training session with the staff conducting the weighing and measuring in schools:

We gave it to [NCMP] staff because they were saying sometimes children can make comments when they’re being weighed that they wanted to follow up on… We felt that it was only right that they were a bit more confident with how to answer those questions or with what to say and how to approach that. (P10)

The same LA also developed a 90 min stand-alone training session for parents or professionals and was currently adapting it for further audiences including for general practitioners (GPs) and practice staff.

Other participants used the guidance in their regular training update for school nurses, with one explaining she emailed it to her team saying, ‘If you’re speaking to a parent and they’re not sure how to talk to their children, email them this document’ (P19). Others talked about including it as a key resource for staff:

It’s been [used] in the electronic resource packs… for school nurses and health and well-being assistants… should they need any help around any topic… It’s in our [healthy weight folder]and people access it as and when. (P22)

Several participants had used the guidance to train staff not directly involved with the NCMP. For example, the guidance was ‘signposted at multiple stages in our [staff] on-boarding’ (P02) in an organisation commissioned to deliver weight management services by a LA. One participant incorporated it into their training for health professionals, noting a GP had commented how it was ‘quite helpful to have that tool, and to have something to be able to give parents as well’ (P20). Another participant had not used it with GPs yet but felt this group might benefit from it:

We get feedback from families [about] how healthy weight was raised from GPs and it’s not always positive. I think they would be a really… key group for this resource to be shared with. (P01)

It was also shared with educational professionals who, though not directly involved in delivering the NCMP, often faced weight-related conversations due to the measurements being taken in school. P15 explained they told schools about the guidance, with their colleague adding:

I think it’s really beneficial for those that aren’t in this line of work, to have something to refer back to that they can use if they’re a little bit unsure… if it’s a staff member at school that’s never really brought up that subject, it’s really useful for them. (P16)

Staff in another LA explained they referred schools to the online e-learning module on the guidance and ‘encourage the schools… to have around by 50% of their staff… complete the training’ (P05). Another participant mentioned sharing it with early years (preschool) staff, explaining, ‘I know it’s not aimed at that age range, but I still think it’s an important one for them to be aware about’ (P09).

### Benefits of using the guidance

Participants frequently spoke of a sense of relief at discovering the guidance, and that it filled a significant gap: ‘I just don’t think there was anything… else like it [that] pulled [advice] together in one place’ (P01). Another participant talked about the guidance generating ‘excitement’ during staff training:

People were really keen and interested and enthusiastic about the document and thought, ‘yeah, this will be brilliant! This is just what parents need, and I’m definitely going to use it and share it.’ (P22)

Participants felt reassured the guidance had been developed by academics and was therefore backed up by research evidence. This increased their confidence in recommending it to parents:

It’s a no brainer, it’s researched, evidence-based, and … it’s just a tool that we’ve got in our tool bag now that we can use for ourselves, or for parents. It’s great. (P19)

The style and tone of the document was also welcomed, with one participant noting that the language fitted with their LA’s approach to weight management:

The language we’re trying to use particularly around NCMP… is that it’s about healthy growth at all sizes and that more compassionate, less stigmatising language. And [having] that in bold at the front of the guide really helps us to say, ‘This isn’t just for you and your family, this is all families.’ (P07)

(Note: this participant is referring to page 2 of the guidance which states ‘This guidance is for parents and caregivers of children of all shapes and sizes’.)

Importantly, many participants talked about how the guidance boosted people’s confidence in talking about a difficult and sensitive subject. One participant felt it *‘*helped remove some of that fear’ (P07) parents have about discussing weight. Another appreciated the practical examples of what to say and how to say it: ‘it’s good where it talks [about] you [can] try this phrase or rephrase it that way’ (P14). Several participants recognised that teachers rarely receive training on how to have these conversations and the guidance could help fill that gap:

I think it’s really beneficial for those that aren’t in this line of work, to have something to refer back to… if they’re a little bit unsure. Because it can be quite challenging. It’s challenging for us, and we’ve done it for years! But if it’s a staff member at school that’s never really brought up that subject, it’s really useful for them. (P16)

Even school health assistants conducting phone calls with parents may not have received training and again the guidance proved useful:

It was a really useful… because it’s exactly what we needed for the phone calls. Because hardly any of us have had training in how to raise the issue of weight because it’s been quite a taboo subject… it was useful to be able to share what wording to use. (P21)

### Limitations of the guidance

While feedback on the guidance was overwhelmingly positive, participants identified some features which might limit the accessibility of the guidance for particular groups. The guidance is 20 pages, which some participants felt was too long for parents with low literacy levels:

When you think that a lot of our children who are of an unhealthy weight, they come from deprived backgrounds, where there is low level literacy skills, parents will struggle then to access it. (P05)

In response, one participant reported developing a shortened version for *‘*parents who will not be able to read that entire document’ (P01).

Others felt it might need to be adapted for use with parents with learning disabilities. One participant explained they might provide these parents with the whole document but would direct them to ‘snippets of the guidance [that]… would work for the family,’ adding the conversation would need to be ‘tailored… to what their understanding would be’ (P14). Additionally, some participants suggested more could be done to make the guidance culturally inclusive:

We have a vast diversity of languages in the borough and lots of our families have a fairly low level of English… so we felt that maybe it could be translated into some key languages …Also some of the imagery wasn’t always reflective of our population. (P13)

Several participants suggested there was a pressing need for similar guidance for parents of teenagers who may be particularly worried about the sensitivities of discussing weight with their children.

If I was to have one ask it would be that the next iteration is targeted at the 13 to 18 age group… [that] would be really powerful, particularly because there’s a lot of misconceptions and … conflicting evidence about the risks of talking to teenagers about weight and the concerns that it generates eating disorders. (P02)

More complex issues about the utility of the guidance were raised by other participants. One participant noted that while a step in the right direction, the guidance on its own was unlikely to change much:

There’s lots of reasons as to why parents wouldn’t want to talk about healthy weight with their kids. And I think the guidance is a really useful tool but I’m not necessarily sure just by looking at that in isolation that would make too much of a difference. (P01)

Several participants also noted in the context of significant deprivation, discussions around weight simply were not a priority for many families:

I’ve given it to parents for them to kind of take away and have a little read but… healthy eating and exercise comes bottom of the list when they’ve got so much on their plate… it’s all those barriers and all those struggles that they’re having and the money issues … [It’s] all those other areas… that need to be addressed first. (P09)

Another felt the guidance was useful but was unsure how it could adequately counter the negative framings encountered in society more generally:

Whilst [the guidance is] absolutely great, how do you manage that against the alternative messages that children and families are getting…[in] social media, marketing, films? (P18)

This concern for consistent messaging was echoed by other participants who felt all the professionals a family might encounter needed to use the same approach:

For me it’s about making sure that all the messaging that goes out to families is coordinated… so that families are receiving the same information from various different sources. (P20)

This participant went on to consider whether parents—particularly those in the most challenging circumstances—could or should be asked to address an issue which she felt was a systemic and environmental issue:

There’s a huge assumption that every parent has the ability and capability to be able to have that conversation in an appropriate and compassionate way with their child…. It puts a lot of responsibility on the parent when actually we deeply believe it’s a system issue and environmental issue… (P20)

## Discussion

This study explored the implementation of a parental guidance document on talking to young children (4–11 years) about weight. Organisations used the guidance in various ways, including providing parents with a link via NCMP letters, mentioning it in follow-up phone calls or incorporating it into training and support for health and education professionals, including GPs and teachers. Participants were positive about the guidance, noting it ‘filled a gap’ by providing an evidence-based and tonally-sensitive resource which increased both their own and parents’ confidence in addressing a sensitive issue. The variation in levels of awareness and use between participants several years after it became available suggests that, while simply including a link to the guidance in template letters may be enough to prompt its use in some areas, it is not enough to embed it consistently across LAs. More work is needed to make the guidance more readily available, and part of standard care, before the reach of the resource is maximised. Potential limitations of the guidance that may prevent wider implementation included its limited suitability for those with lower literacy levels, the need for additional parental (and professional) guidance on discussing weight with adolescents and recognition that the guidance was just one small part of a much-needed systems-wide approach to tackling overweight and obesity.

The perceived usefulness and relevance of the guidance aligns with and extends initial pilot findings^11^ and supports the need to extend implementation of the guidance at scale. Participants considered the guidance trustworthy, reassured by its clear link to an evidence base. While DHSC guidance^25^ is available for healthcare professionals in the form of a ‘conversation framework’ for talking to parents about the NCMP (issued 2019, updated 2023), this does not provide guidance on talking to *children* and is not suitable for sharing with parents. It was notable that no participants referred to this partner guidance document in the present study, despite its availability since 2019, suggesting they lack awareness of this resource too.

Barriers to using the guidance included its potential inaccessibility for families facing greater deprivation, from ethnic minority cultural backgrounds or with lower educational levels. Some PHPs did not share the guidance with these groups, which could result in a two-tier system with the potential to widen inequalities in access to support. Our public contributors felt increasing access to the guidance in these more marginalised communities should be a specific priority for commissioners.

Participants recognised the tension between helping parents to address children’s weight while avoiding increasing feelings of blame. Providing the guidance to all parents, regardless of their child’s weight, may help reduce perceptions of stigma. Participants mostly endorsed this approach, noting the guidance is relevant for all parents, as even healthy weight children may worry about their weight or encounter potentially harmful weight-related content online. In past work,^11^ parents and school nurses suggested introducing the guidance in advance of the NCMP, giving parents time to address their own and their child’s emotional responses and concerns about the measurement process. However, parents may not find it relevant until they receive specific information about their child.^11^ This suggests use of the guidance may be enhanced by both embedding it into invitation letters and operational guidance to reduce gatekeeping on the part of practitioners, as well as making it widely available to parents and health, social care and education practitioners working with children and families.

Many interviewees found the guidance useful for other elements of their role, such as in children’s weight management services, and in links with schools and primary care, ensuring a common approach and language. Individuals are more likely to trust and adopt advice from health and care professionals when it is consistent,^26^ endorsing a case for wider distribution to professionals beyond the NCMP. In a recent study with GPs in Ireland, the guidance was perceived as useful in overcoming barriers to talking to families about children’s weight, avoiding stigma and providing a framework for conversations.^27^ They also identified a need for advice to be consistent across multidisciplinary teams. Our findings also suggest school staff may benefit from the resource, with our public contributors emphasising the importance of teachers and other school employees due to their daily interaction with children, and because NCMP measurements take place within schools.

### Limitations

While this study offers valuable insights into the importance PHPs place on the guidance and how it is currently being used, we acknowledge several limitations. First, in line with the finding of variable awareness and uptake across LAs, one participant had only recently become aware of the guidance and had not yet applied it in practice, while others reported just beginning to use it. As a result, we are only able to capture the early stages of its adoption and acknowledge its use in practice may evolve over time. Related to this—and noting the exception mentioned above—those unaware of the guidance did not volunteer for interviews, meaning we cannot provide insights into the broader barriers to awareness and implementation. Given the relatively small number of interviewees, we were unable to sample for geographical and occupational diversity. There were no interview participants from the North-East, East and South-East of England. Additionally, we are aware that the findings reflect a UK-based programme, and further research should look at similar measurement programmes in Europe,^28^ as well as non-mandated measurement experiences in the USA,^29^ Canada^30^ and Australia.^31^

Finally, interview participation depended on whom we reached within organisations, with responses inevitably influenced by factors like role, time in position and personal views, as well as reflecting organisational approach. In some areas where we knew the guidance had been used extensively, staff changes meant we were unable to capture past implementation practices through our methods. Some participants referred to procedures or training suggesting the guidance had been embedded within standard practice; however, others responded based on their own experience and use, rather than representing policy within a given organisation.

### Implications for research and practice

This study, together with past work,^11 27^ indicates the guidance is useful, credible and relevant to professionals and parents, and fills a gap that is not met by other resources. In several areas, staff have used the guidance to develop their own training and initiatives involving schools and parents, suggesting that the guidance has practical application in clinical, community and educational settings. Further research is needed to explore the impact of the guidance on parents and children, and if/how it promotes children’s well-being and healthy weight.^16^

The guidance appears to help PHPs engage with parents in discussions about childhood weight in a timely and appropriate way, potentially reducing avoidance of this sensitive topic. Broader health research suggests having an accessible resource to share with patients about their conditions can increase practitioners’ confidence in raising the topic.^32 33^ Our earlier work^11^ has also found that parents find the guidance more useful when they can discuss it with a practitioner, highlighting that professional acceptability and confidence are central to wider parental uptake. More broadly, our findings underline the importance of consistency in how healthy child weight is discussed with families across disciplinary and organisational settings. This study has found the guidance is already being adopted beyond the NCMP into community healthy weight services, GP practices and educational settings, and may help ensure greater alignment in language, tone and approach across these different settings.

### Recommendations to increase impact

Despite its potential, the impact of the guidance is currently limited by variable awareness, inconsistent embedding into core processes and lack of application to other groups. To enhance its reach and impact, we recommend:

1. Stronger endorsement and branding of the guidance by DHSC to allow systematic distribution and integration into operational guidance.
2. Sharing best practice on how the guidance could be used—for example: training for NCMP staff, multidisciplinary teams and school staff; providing links in both NCMP invitation and results letters; and within phone conversations with families.
3. Adapting the guidance for different groups, including shorter or pictorial versions for families with lower literacy levels, translation into different languages and tailored versions for families with learning disabilities. Since completing this research, a video version of the guidance has been created (forthcoming). Separate work is currently underway to develop guidance for parents of adolescents with age-appropriate additional content and framing.

## Conclusions

Public health professionals viewed guidance for parents on talking to children about weight as a valuable tool for staff training and engaging with families. However, the guidance had not yet been embedded within core NCMP procedures and participants identified barriers to its wider implementation. To maximise impact, the guidance should be made available in more accessible formats, and more widely disseminated and applied across the different pathways through which healthy weight may be raised with families (eg, NCMP, community healthy weight services, GPs and schools). Embedding the guidance within a system-wide approach will help ensure children and families receive a consistent, evidence-based and supportive discussion about this sensitive topic.

## Supplementary material

10.1136/bmjopen-2025-105371 online supplemental file 1

10.1136/bmjopen-2025-105371 online supplemental file 2

## Data Availability

Data are available upon reasonable request.
