# Supplementary material for: How public health practitioners in the UK are using parental guidance on talking to children about weight: a qualitative study
Source: BMJ Open. 2026 Feb 25;16(2):e105371. doi: 10.1136/bmjopen-2025-105371 (PMC12959042; doi:10.1136/bmjopen-2025-105371)
Supplement: online supplemental file 1 [file bmjopen-16-2-s001.docx]

Additional file 1

Topic guide for local authority staff

**Delivery of the ‘Talking to your child about weight’ guidance for parents: interview schedule**

*<Introduction about the project and aims for the interview, in line with information sheet>*

1. Please tell us more about your role within the local authority, and the contact you have with families around healthy weight.
2. How did you first find out about the guidance?
3. What did you make of the guidance when you first saw it?
4. How did it differ from or add to what you were drawing on previously?
5. Could you tell us how it is included in how you deliver the NCMP or other support to families now?
6. Did you always use the guidance in this way, or have you tried other ways previously?
   1. What do you think are its strengths and limitations when used in this way?
7. Do you plan to make any changes next school year in how you use the guidance?
   1. If so, what?
8. Do you have any specific examples you can share of when the guidance has been helpful?
9. Have you used the guidance with families from different socio-economic or cultural backgrounds?
   1. If yes, please tell us about any differences or adaptations needed
10. What barriers have you encountered to using this guidance?
11. If we were doing a formal evaluation of what effect the guidance has on families and local authority public health practitioners like you, what should we measure?
    1. What advice would you offer us on how to do this?
12. The reason we introduced the guidance was to try and reduce parent uncertainty and anxiety about whether and how to talk to their child about weight, if and when they need to, and to improve children’s experiences of talking about weight at home.

- 1. How well do you think the guidance meets these aims?
  2. What other needs do you think parents have around talking positively to children around a healthy weight?
  3. What other ways does your team support parents in having these conversations as and when they need to?

1. Thank you for your time in talking to us. Is there anything else you’d like to add on this topic?
