# Supplementary material for: How public health practitioners in the UK are using parental guidance on talking to children about weight: a qualitative study
Source: BMJ Open. 2026 Feb 25;16(2):e105371. doi: 10.1136/bmjopen-2025-105371 (PMC12959042; doi:10.1136/bmjopen-2025-105371)
Supplement: online supplemental file 2 [file bmjopen-16-2-s002.docx]

**Supplementary file - COREQ Statement**

How public health practitioners in the UK are using parental guidance on talking to children about weight: A qualitative study

**Domain 1: Research team and reflexivity**

*Personal Characteristics*

1. Interviewer/facilitator. Which author/s conducted the interview or focus group?

*Rowan Brockman*

2. Credentials. What were the researcher’s credentials? E.g. PhD, MD.

*PhD*

3. Occupation. What was their occupation at the time of the study?

*Senior Research Associate*

4. Gender. Was the researcher male or female?

*Female.*

5. Experience and training. What experience or training did the researcher have?

*18 years’ experience as a qualitative researcher. Extensive training in qualitative research methods (PhD and subsequent courses).*

*Relationship with participants*

6. Relationship established. Was a relationship established prior to study commencement?

*No direct relationship was established with the research participants prior to recruitment.*

7. Participant knowledge of the interviewer. What did the participants know about the researcher? e.g. personal goals, reasons for doing the research?

*Participants were sent a Participant Information Sheet (PIS) which set out the purpose of the study, why the participant have been asked to take part and what the interview would entail. This information was also repeated verbally before the start of the interview. The study Principal Investigator and co-investigators were named on the PIS and the interviewer (RB) introduced herself to participants as a researcher working at the University of Bristol at the start of the interview.*

8. Interviewer characteristics. What characteristics were reported about the interviewer/facilitator? e.g. Bias, assumptions, reasons and interests in the research topic.

*We have reported that the researcher/interviewer is an experienced qualitative researcher (page 8). The interviewer is a qualitative researcher with many years’ experience conducting qualitative research on a range of health topics. She had not previously worked on projects involving the National Child Measurement programme but have previously worked on studies involving weight management and physical activity in children.*

**Domain 2: study design**

*Theoretical framework*

9. Methodological orientation and Theory. What methodological orientation was stated to underpin the study? e.g. grounded theory, discourse analysis, ethnography, phenomenology, content analysis

*In the Methods section we explain how we used thematic analysis with NVivo12 software. This is an established methodology underpinned by an interpretative epistemological perspective (page 8).*

*Participant selection*

10. Sampling. How were participants selected? e.g. purposive, convenience, consecutive, snowball.

*We recruited a convenience sample (described on page 7).*

11. Method of approach. How were participants approached? e.g. face-to-face, telephone,

mail, email.

*Participants were recruited via through multiple channels, as described on page 7. This included emailed updates via OHID and other personal networks, and a webinar hosted by OHID. Participants could contact the researchers directly to arrange an interview. All interviewees gave informed verbal consent before the start of the interview.*

12. Sample size. How many participants were in the study?

*There were 24 participants, participating in 16 interviews (some were joint interviews).*

13. Non-participation. How many people refused to participate or dropped out? Reasons

*Two people expressed interest in participating but subsequently cancelled their appointments. We contacted them to reschedule twice but received no response.*

14. Setting of data collection. Where was the data collected? e.g. home, clinic, workplace

*Interviews were conducted online using Teams.*

15. Presence of non-participants. Was anyone else present besides the participants and researchers?

*No-one else was present besides the interviewer and participant(s).*

16. Description of sample. What are the important characteristics of the sample? e.g. demographic data, date.

*Participants were all professionals working in child weight management and NCMP delivery. Sample characteristics are reported in Table 1 on page 9/10 and described on page 11.*

*Data collection*

17. Interview guide. Were questions, prompts, guides provided by the authors? Was it pilot

tested?

*We used a broad topic guide focusing on: participants’ awareness of the guidance, if/how it was used, perceived impact on parents, plans for future use, and relevance for their local population. The topic guide was created with input from all authors; it was not pilot tested before use. The topic guide is provided in Additional file 1.*

18. Repeat interviews.

*There were no repeat interviews.*

19. Audio/visual recording. Did the research use audio or visual recording to collect the data?

*All interviews were audio recorded, with the informed consent of participants (page 8).*

20. Field notes. Were field notes made during and/or after the interview or focus group?

*Field notes were not made during this study.*

21. Duration. What was the duration of the interviews or focus group?

Interviews lasted 23-62 minutes (page 8).

22. Data saturation. Was data saturation discussed?

*We interviewed all available participants. We discussed whether we needed to have another round of recruitment to increase the sample. However, drawing on the concept of information power rather than data saturation we felt we had sufficient and appropriate levels of information power for our research objectives.*

23. Transcripts returned. Were transcripts returned to participants for comment and/or correction?

*No.*

**Domain 3: analysis and findings**

*Data analysis*

24. Number of data coders. How many data coders coded the data?

*Data were coded by the researcher RB following the development of the coding frame which was developed by RB and RL following detailed examination of a sample of transcripts. Coding and iterative developments were discussed at regular meetings (page 8).*

25. Description of the coding tree. Did authors provide a description of the coding tree?

*No.*

26. Derivation of themes. Were themes identified in advance or derived from the data?

*Themes were derived deductively from research questions and inductively from participant responses.*

27. Software. What software, if applicable, was used to manage the data?

*NVivo 12 qualitative software.*

28. Participant checking. Did participants provide feedback on the findings?

*No. We discussed our results with our public contributors.*

29. Quotations presented. Were participant quotations presented to illustrate the themes /

findings? Was each quotation identified? e.g. participant number

*Quotations are used to illustrate themes. Quotes are labelled as P(number) (for interview participant) (Page 8)*

30. Data and findings consistent. Was there consistency between the data presented and the findings?

*Yes.*

31. Clarity of major themes. Were major themes clearly presented in the findings?

*Yes (pages 12-18)*

32. Clarity of minor themes. Is there a description of diverse cases or discussion of minor themes?

*Yes (e.g., pages 12-14 describe the different ways guidance was being used and page 18 discusses differing views on the utility of the guidance.)*
