# Supplementary material for: How public health practitioners in the UK are using parental guidance on talking to children about weight: a qualitative study
Source: BMJ Open. 2026 Feb 25;16(2):e105371. doi: 10.1136/bmjopen-2025-105371 (PMC12959042; doi:10.1136/bmjopen-2025-105371)
Supplement: Supplementary file 3 [file bmjopen-2025-105371.reviewer_comments.pdf]

PEER REVIEW HISTORY

BMJ Open publishes all reviews undertaken for accepted manuscripts. Reviewers are asked to complete a checklist review form and are provided with free text boxes to elaborate on their assessment. These free text comments are reproduced below.

ARTICLE DETAILS

Title (Provisional)

How public health practitioners in the UK are using parental guidance on talking to children about weight: A qualitative study

Authors

Brockman, Rowan; Gillison, Fiona; Grey, Elisabeth B; Jago, Russ; MacArthur, Georgie J; Gutteridge, Callum; Langford, Rebecca

---

VERSION 1 - REVIEW

**Reviewer**	**1**
**Name**	**Gomes, Ana Isabel**
**Affiliation**	**University of Lisbon Faculty of Psychology**
**Date**	**07-Aug-2025**
**COI**	**None**

Reviewer's comments

Thank you for the opportunity to review this manuscript, entitled "How are professionals using parental guidance on talking to children about weight? A mixed-methods study" (bmjopen-2025-105371). The present work aims to provide information about how health practitioners are using the "Talking to my child about weight" guide. The study presents interesting results that clarify how and why health professionals use this parental guide in conversations about children's weight, their perception of the quality and usefulness of the available documentation, and the guide's dissemination range, which contributes to a more informed discussion about potential needs for improvement. However, I have some concerns while reading this manuscript, which I share below.

My first concern is regarding the designation of the study as a mixed-methods study. Although the study includes two different types of instruments (a questionnaire with open- and closed-ended questions, and an interview), in my opinion, there does not appear to be a true blending ("mixing") or integration of the methods and/or data obtained (for example, as occurs in an explanatory sequential design), which is mandatory in such a research. The authors do not clarify which specific research design was used within mixed methods research, nor does the explanation throughout the manuscript clarify this point, which does not help to clarify this issue. For instance, in the introduction, the authors justify using mixed-methods research with the work's aim, without clarifying why this aim could only (or better) be answered with this type

of research. There also appears to be some overlap between the quantitative and qualitative questions posed in both instruments and the evidence obtained, which is not sufficiently synthesized and combined. I suggest consulting and following, in addition to the reporting guidelines for qualitative and quantitative studies used, a specific guideline for mixed-methods research (e.g., JARS-Mixed), to report the different phases of the study and the methodological choices in a more complete way, if the authors decide to maintain this study design designation.

I have other concerns regarding some of the authors' methodological choices and procedures reported regarding data collection and analysis:

- The authors explain throughout the article that they encountered recruitment issues, which fell short of their expectations. Understandably, this led the authors to take some measures; however, these measures must be justified to ensure transparency regarding the data's source and credibility. For example, if the study's objective is to understand how the guidance is used in pediatric services and explore barriers and facilitators to its implementation, does it make sense to include participants regardless of their prior or current use of the guidance (p. 7)? What impact might this have had on the richness of data collected and on answering the research question? The sampling method should also be in the data collection section.

- It is not clear why the interview script followed "similar topics to the survey (l. 129)"; what is the advantage? Issues and specific cautions regarding interviewing more than one person at a time should be addressed. Since the study collected data through interviews, the authors should also include the COREQ checklist. There is relevant information missing in the manuscript (e.g., interviewer characteristics, experience and training, previous relationship with participants, participant knowledge of the interviewer, ethical procedures).

- The way participants are identified in the quotes is unconventional and somewhat confusing to the reader. I think it would have made more sense to assign a number to each participant and then indicate the S and/or P in front of them, if they responded to both questionnaires. I also think it is important to understand which other characteristics might be relevant to mention in this identification (e.g., job title, NCMP vs no-NCMP).

- The manuscript should also address, throughout the text or in a specific section, information regarding methodological integrity.

I also have some questions and comments about presentation of results:

- The information regarding the Survey participants (p. 9) is not exclusively regarding the survey. For instance, in Table 1, the authors provided information about participants who did the interview. Since not all participants completed the survey and answered the interview, and some interviews were performed with more than one person, it could be useful to present a flow diagram to achieve a global perspective on data origin and changes in numbers due to attrition.

- Despite all the limitations and issues highlighted above, related to participant selection and eligibility criteria, the qualitative analysis is the strongest part of the manuscript (the information collected is relevant; the themes and quotes are well organized). Nevertheless, there is some overlapping information regarding what was collected in surveys, regarding, for instance, how the guidance is used and provided to parents (p. 12). This makes me wonder again whether the survey data introduces any new and/or relevant information to the data collected in the interview. I suggest additional efforts in synthesizing the qualitative/quantitative data presented.

Regarding the discussion:

I think the authors also did a good job in the discussion, seeking to reflect on and frame information they weren't expecting to receive, and the people who, despite being direct participants in the work carried out by the team of authors, didn't make themselves available to be interviewed. I agree with the authors that the mixed study that was intended to be carried out was not effectively accomplished in these terms. However, as I mentioned earlier, I do not think that we can put all the blame on recruitment problems.

Nevertheless, it seems to me that the authors could reflect more on what went less well in the dissemination of the materials produced and training about how they should be used, and how this can be overcome in the future, to ensure that users understand the guide's potential and limitations and that they use it appropriately with their parents. I think it is also important to remember that a considerable percentage of participants interviewed did not collaborate in NCMP, did not receive training, and/or do not use it regularly to guide their practice, which limits the ability to draw deeper conclusions from the data collected. Finally, the authors rightly mention that it is important for families to first receive information about their child, so they can decide whether or not to consult the guide. Regarding this point, it may be interesting to mobilize some literature on the role of risk perception as a driver of information seeking and behavior change, considering this population and this intervention context.

**Reviewer**	**2**
**Name**	**Petrelli, Fabio**
**Affiliation**	**Universita degli Studi di Camerino**
**Date**	**30-Aug-2025**
**COI**	**None**

Title, not full clear. I am not a fan of question marks in titles. I suggest clarifying this aspect and specifying the relevant care setting in the title;

Abstract, please consider to update the conclusion section in clinical practice view (see the comments in the discussion);

Keywords, please consider the same suggestion for clinical setting (Country-Region);

Introduction: I suggest to improving this section in clinical practice consideration. The aims aren't full clear; please adopt classical formula as "the primary aim was.. while secondary...";

Methods: well structured but I don't see clear reference in the text of the correct reporting tool adopted and reported in the supplemenentary files; missing references of the tool in the dedicated section;

Results: Good purpose that not required relevant update.

Discussion: I suggest to reconsider the section. It's starting with the discussion with a limit and not with data finding in international view. Please consider a multi-disciplinary and organizational perspective that are lacking. In this regard, I suggest that the authors include

a dedicated section entitled 'Perspective for Clinical Practice', which could start from the discussion of topics such as 'Achieving quadruple aim goals through clinical networks' and 'Perspectives for Primary Health Care public policy', leading to the necessary organizational connections based on the collected data. In addition to completing the section more effectively, addressing this gap will be essential in the next version of the manuscript.

Limits: not recommended the use of references in this section. I suggest, if possible, the references in the discussion. Please poned major attention to generalizabilty of data finding for population and not only in the tool adopetd.

Conlusion: update according previous corrections;

References: not always up-to-date (some over ten years old) and will require necessary updating, also following the provided indications.

VERSION 1 - AUTHOR RESPONSE

Reviewer 1

Dr. Ana Isabel Gomes, University of Lisbon Faculty of Psychology

	Reviewer comment	Author response
1	My first concern is regarding the designation of the study as a mixed-methods study. Although the study includes two different types of instruments (a questionnaire with open- and closed-ended questions, and an interview), in my opinion, there does not appear to be a true blending ("mixing") or integration of the methods and/or data obtained (for example, as occurs in an explanatory sequential design), which is mandatory in such a research. The authors do not clarify which specific research design was used within mixed methods research, nor does the explanation throughout the manuscript clarify this point, which does not help to clarify this issue. For instance, in the introduction, the authors justify using mixed-methods research with the work's aim, without clarifying why this aim could only (or better) be answered with this type of research. There also appears to be some overlap between the quantitative and qualitative questions posed in both instruments and the evidence obtained, which is not sufficiently synthesized and combined. I suggest consulting and following, in addition to the reporting	Comment no longer relevant after removal of survey data.

	guidelines for qualitative and quantitative studies used, a specific guideline for mixed-methods research (e.g., JARS-Mixed), to report the different phases of the study and the methodological choices in a more complete way, if the authors decide to maintain this study design designation.	
2	The authors explain throughout the article that they encountered recruitment issues, which fell short of their expectations. Understandably, this led the authors to take some measures; however, these measures must be justified to ensure transparency regarding the data's source and credibility.<br><br>For example, if the study's objective is to understand how the guidance is used in paediatric services and explore barriers and facilitators to its implementation, does it make sense to include participants regardless of their prior or current use of the guidance (p. 7)? What impact might this have had on the richness of data collected and on answering the research question?<br><br>The sampling method should also be in the data collection section.	The first part of this comment largely relates to the low response rate for the survey and is therefore no longer relevant after removing this aspect of the study.<br><br>We allowed participants to take part regardless of their prior or current use of the guidance as we wanted to understand potential barriers to its use. Only one participant had not yet used the guidance in their practice – this has been noted in the limitations section.<br><br>We explain in the Participants and recruitment section of the Methods that we interviewed all participants who expressed an interest. We note in the limitations we were unable to sample for geographical or occupational diversity.
3	It is not clear why the interview script followed "similar topics to the survey (l. 129)"; what is the advantage?	Comment no longer relevant after removal of survey data.
4	Issues and specific cautions regarding interviewing more than one person at a time should be addressed.	We have now added text to the Data Collection section within the Methods, discussing the benefits and pitfalls of using more than one participant in interviews, with references which explore this further:<br><br>*"Whilst there are notable disadvantages to dyadic interviews, such as sensitive disclosures being inhibited due to a colleague's presence and the relational nature of the data making coding and interpretation more complex, they have distinct advantages resulting from participants having a shared interest in the topic*

		and a pre-existing relationship between them. For example, the presence of two participants allows them to build on or challenge each other's accounts, creating more layered and nuanced narratives than a single interview might produce. Additionally, dyads can reproduce natural conversation patterns, making the data feel more authentic and reflective of real-world interaction (Morgan et al., 2013; Eisikovits & Koren, 2010; Szulc & King, 2022; Wimbauer & Motakef, 2017)" [p.8]
5	Since the study collected data through interviews, the authors should also include the COREQ checklist. There is relevant information missing in the manuscript (e.g., interviewer characteristics, experience and training, previous relationship with participants, participant knowledge of the interviewer, ethical procedures).	The COREQ checklist is included in the submission as a separate file, but we have now added brief details of the relevant information to the manuscript, as suggested:<br><br>*"Interviews were conducted online between February-May 2024 by RB, allowing her to develop familiarity and a comprehensive view of the dataset before analysis. RB is a female, postdoctoral researcher with 18 years of experience in qualitative research on a range of health topics, including weight management and physical activity in children. Participants had no prior knowledge or previous relationship with the interviewer."* [p.8]<br>We have also amended the text in the Ethical Approval section of the Methods to state that participants were provided with a Participant Information Sheet prior to interview:<br><br>*"We provided a Participant Information Sheet and obtained verbal consent from participants before interviews"*. [p.9]
6	The way participants are identified in the quotes is unconventional and somewhat confusing to the reader. I think it would have made more sense to assign a number to each participant and then indicate the S and/or P in front of them, if they responded to both questionnaires.	Having removed the survey element of the study we have simplified the participant identifiers.
7	I also think it is important to understand which other characteristics might be relevant to mention in this identification (e.g., job title, NCMP vs no-NCMP).	Having removed the survey element, we have revised Table 1 (Characteristics of interview participants) which now includes a broad description of participants' job roles, whether participants were interviewed jointly, and how the NCMP guidance was used. Specific job

		titles were not included to avoid possible identification.
8	The manuscript should also address, throughout the text or in a specific section, information regarding methodological integrity.	Thank you for this comment. Though we already discuss issues relating to methodological integrity within the methods section, we have enhanced this by noting the benefit of having a single interviewer in developing a holistic view of the dataset before starting analysis, a new paragraph reflecting on dyadic interviews, and how analysis was led by RB but in regular discussion with RL, the study team and our public contributors. [Please see Data Collection and Data Analysis sections within Methods, pp. 8-9].<br><br>We have also added relevant information about the interviewer's background and relationship with participants from the COREQ checklist (which is provided as a separate file) to enhance the methodological integrity<br><br>"RB is a female postdoctoral researcher with 18 years of experience in qualitative research on a range of health topics, including weight management and physical activity in children. Participants had no prior knowledge or previous relationship with the interviewer prior to recruitment." [p.8].
9	The information regarding the Survey participants (p. 9) is not exclusively regarding the survey. For instance, in Table 1, the authors provided information about participants who did the interview. Since not all participants completed the survey and answered the interview, and some interviews were performed with more than one person, it could be useful to present a flow diagram to achieve a global perspective on data origin and changes in numbers due to attrition.	Comment no longer relevant after removal of survey data.
10	Despite all the limitations and issues highlighted above, related to participant selection and eligibility criteria, the qualitative analysis is the strongest part of the manuscript (the information collected is relevant; the themes and quotes are well organized). Nevertheless, there is	Comment no longer relevant after removal of survey data.

	some overlapping information regarding what was collected in surveys, regarding, for instance, how the guidance is used and provided to parents (p. 12). This makes me wonder again whether the survey data introduces any new and/or relevant information to the data collected in the interview. I suggest additional efforts in synthesizing the qualitative/quantitative data presented.	
11	Regarding the discussion: I think the authors also did a good job in the discussion, seeking to reflect on and frame information they weren't expecting to receive, and the people who, despite being direct participants in the work carried out by the team of authors, didn't make themselves available to be interviewed. I agree with the authors that the mixed study that was intended to be carried out was not effectively accomplished in these terms. However, as I mentioned earlier, I do not think that we can put all the blame on recruitment problems.	Comment no longer relevant after removal of survey data.
12	Nevertheless, it seems to me that the authors could reflect more on what went less well in the dissemination of the materials produced and training about how they should be used, and how this can be overcome in the future, to ensure that users understand the guide's potential and limitations and that they use it appropriately with their parents.	We have now re-drafted the 'Limitations' section and added in text relating to differences in implementation practices between organisations:<br><br>*"While this study offers valuable insights into how the guidance is currently being used, we acknowledge several limitations. One participant had only recently become aware of the guidance and had not yet applied it in practice, while others reported just beginning to use it. As a result, we are only able to capture the early stages of its adoption and acknowledge its use in practice may evolve over time."* [p.27]<br><br>We now include revised sections on implications for research and practice and recommendations to increase impact [pp. 28-29].
13	I think it is also important to remember that a considerable percentage of	All bar one of our interviewees (n=23) had used the guidance in some capacity, whether

	participants interviewed did not collaborate in NCMP, did not receive training, and/or do not use it regularly to guide their practice, which limits the ability to draw deeper conclusions from the data collected	that were in training, education or practice. The one interviewee who was aware of the guidance but hadn't used it was a survey respondent. We have amended the text in the 'Limitations' section to reflect the fact that some interviewees had only recently become aware of the guidance, which would affect the depth of conclusions drawn from the study.<br><br>In response to the Reviewer's point about the lack of training, while this is the case, there is no intended further training or central call on practitioners to use the guidance. As such, we believe one of the functions of this study is to highlight that the approach to dissemination taken by DHSC so far results in limited uptake, and leads to our call for greater attention to how this resource may be better embedded into standard use.<br><br>*"While this study offers valuable insights into how the guidance is currently being used, we acknowledge several limitations. One participant had only recently become aware of the guidance and had not yet applied it in practice, while others reported just beginning to use it. As a result, we only able to capture the early stages of its adoption and acknowledge its use in practice may evolve over time. Related to this – and noting the exception mentioned above – those unaware of the guidance did not volunteer for interviews, meaning we cannot provide insights into the broader barriers to awareness and implementation* [p.27].
14	Finally, the authors rightly mention that it is important for families to first receive information about their child, so they can decide whether or not to consult the guide. Regarding this point, it may be interesting to mobilize some literature on the role of risk perception as a driver of information seeking and behavior change, considering this population and this intervention context.	Thank you for this suggestion. After consultation with our co-authors, we feel that a discussion of risk perception as a driver of information-seeking and behaviour change in parents is beyond the scope of this paper. Our focus is primarily on how practitioners are using the guidance with parents, rather than how parents interpret and use the information. Additionally, the guidance aims to reduce parents' perceptions of the risk associated with talking to children about weight (and the potential harm if done incorrectly rather than drive information-seeking behaviour through emphasising risk.

Reviewer: 2
Dr. Fabio Petrelli, Universita degli Studi di Camerino

	Reviewer comment	Author response
1	Title, not full clear. I am not a fan of question marks in titles. I suggest clarifying this aspect and specifying the relevant care setting in the title	Thank you for your comment. We have now revised the title considering these comments, to be "How public health practitioners in the UK are using parental guidance on talking to children about weight: A qualitative study".
2	**Abstract,** please consider to update the conclusion section in clinical practice view (see the comments in the discussion);	Given the abstract's word limit, we believe its conclusion accurately reflects the overall findings of the paper.
3	**Keywords** please consider the same suggestion for clinical setting (Country-Region);	Key words are listed in the online system (rather than the manuscript itself) and include the UK as the country focus. For reference, the keywords included are: Parents, Obesity, Overweight, Child, Qualitative Research, Public Health.
4	**Introduction**: I suggest to improving this section in clinical practice consideration. The aims aren't full clear; please adopt classical formula as "the primary aim was.. while secondary...";	Our study had two keys aims but as both were equally important, we do not feel it is appropriate to express these as primary/secondary aims. We have re-written this section to make the two aims clearer:<br><br>*"This qualitative study had two key aims:*<br>*1. to understand if and how the guidance is currently used in child weight management services*<br>*2. to explore barriers and facilitators to its implementation."* [p.6]
5	**Methods**: well- structured but I don't see clear reference in the text of the correct reporting tool adopted and reported in the supplementary files; missing references of the tool in the dedicated section;	We have now removed the survey element of the paper meaning this is now a purely qualitative paper. We have included the COREQ reporting tool for qualitative research as an additional file and reference this in the manuscript.<br><br>*"The COREQ checklist is provided as Additional File 2."* [p.9].
6	Results: Good purpose that not required relevant update.	Thank you.

7	Discussion: I suggest to reconsider the section. It's starting with the discussion with a limit and not with data finding in international view. Please consider a multi-disciplinary and organizational perspective that are lacking. In this regard, I suggest that the authors include a dedicated section entitled 'Perspective for Clinical Practice', which could start from the discussion of topics such as 'Achieving quadruple aim goals through clinical networks' and 'Perspectives for Primary Health Care public policy', leading to the necessary organizational connections based on the collected data. In addition to completing the section more effectively, addressing this gap will be essential in the next version of the manuscript.	We have now revised the Discussion section so that it does not begin with a limitation [p. 24].<br><br>As the guidance is primarily being used by staff operating in community or school-based settings, the term 'clinical practice' does not feel appropriate. However, we agree it is important to link our findings to specific recommendations for policy and practice. We now include a section called 'Implications for research and practice' where we discuss more explicitly the importance of the guidance in providing a consistent tone, language and approach across disciplinary and organisational contexts, e.g. health, community and educational settings [pp. 28-29].
8	**Limitations**: i) not recommended the use of references in this section. I suggest, if possible, the references in the discussion. ii) Please pay major attention to generalizability of data finding for population and not only in the tool adopted.	i) To provide context and allow readers to explore comparable measurement programs in other countries, we believe it is important to retain references to these international programs in this section.<br>ii) We have now added an 'Implications for research and practice' section discussing the relevance of the tool for practice across healthy weight, general practice and educational settings. We now include the following text:<br><br>*"Broader health research suggests having an accessible resource to share with patients about their conditions can increase practitioners' confidence in raising the topic (Public Health England, 2021; Jiang, Zhao, Tang et al., 2024). Our earlier work (Baber, Gillison & Grey, 2023) has also found that parents find the guidance more useful when they can discuss it with a practitioner, highlighting that professional acceptability and confidence are central to wider parental uptake. More broadly, our findings underline the importance of consistency in how healthy child weight is discussed with families across disciplinary and organisational settings. This study has found*

		the guidance is already being adopted beyond the NCMP into community healthy weight services, GP practices, and educational settings, and may help ensure greater alignment in language, tone and approach across these different settings" [pp. 28-29].
9	**Conclusion:** update according to previous corrections	We have now revised the conclusion in light of the revisions described above.<br><br>*"To maximise impact, the guidance should be more widely disseminated and applied across the different pathways through which healthy weight may be raised with families (e.g. NCMP, community healthy weight services, GPs and schools). Embedding the guidance within a system-wide approach will help ensure children and families receive a consistent, evidence-based and supportive discussions about this sensitive topic"* [p. 30].
10	**References** not always up-to-date (some over ten years old) and will require necessary updating, also following the provided indications.	Thank you for this comment. We only cite two papers older than 10 years (Statham et al, 2011 and Gillison et al. 2014). Both are directly relevant to this paper, referring to parental concerns about the National Child Measurement Programme. We therefore feel it is important to include these papers.

VERSION 2 - REVIEW

**Reviewer**	**1**
**Name**	**Gomes, Ana Isabel**
**Affiliation**	**University of Lisbon Faculty of Psychology**
**Date**	**26-Dec-2025**
**COI**	

I compliment the authors and congratulate them on the significant improvements in the article. The authors properly addressed the reviewers' concerns and the questions raised, and reformulated the description of participant characteristics, the methodological procedures, the discussion and limitations. I have a few final minor suggestions:

Manuscript

Abstract. “To evaluate how public health practitioners are using parental guidance on talking to children in their work with parents.” The authors might want to consider changing the sentence action verb to better align with the choice of performing a qualitative study design to answer this research question: understand or examine. Or instead, adjust the objectives in the abstract to match the latest version of the manuscript.

l.101, p.6: “We used a qualitative study design (interviews) with practitioners working in child weight management and NCMP delivery.” The authors might want to mention, besides the research design and data-collection strategies, provide the rationale for the design selected.

l.116, p.7: “Interviews were conducted online between February-May 2024 by RB, allowing her to develop familiarity and a comprehensive view of the dataset before analysis RB is a female. A point (.) is missing between the two sentences (before RB).

l.169, p.10/11: Table 1. Table 1 presents detailed information about the participants; however, it seems somewhat lengthy to include in the body of the article. I leave it to the authors and the journal editor to consider allocating this table to supplementary material, with the presentation of a summary table indicating the absolute/relative frequencies for each response category.

l.332, p.20 “sensitive resource which increased both their own and parents’ confidence in addressing a sensitive issue The”. Please, add a point before the The.

Supplementary file – COREQ Statement

Please, change the title according to the last version of the manuscript.

Use a single way to refer to a page (page or p).

Section 17. Interview guide. Were questions, prompts, guides provided by the authors? Was it pilot tested?,: It appears question in blue still needs to be answered.

VERSION 2 - AUTHOR RESPONSE

Reviewer 1
Dr. Ana Isabel Gomes, University of Lisbon Faculty of Psychology

I compliment the authors and congratulate them on the significant improvements in the article. The authors properly addressed the reviewers' concerns and the questions raised, and reformulated the description of participant characteristics, the methodological procedures, the discussion and limitations.

We thank the reviewer for her positive remarks.

I have a few final minor suggestions:

Manuscript		
	**Reviewer Comment**	**Author Response**

**1**	Abstract. “To evaluate how public health practitioners are using parental guidance on talking to children in their work with parents.” The authors might want to consider changing the sentence action verb to better align with the choice of performing a qualitative study design to answer this research question: understand or examine. Or instead, adjust the objectives in the abstract to match the latest version of the manuscript.	Thank you for the suggestion, we have now changed the word ‘evaluate’ to ‘understand’ in the Abstract (line 22), to better align with the qualitative study design.
**2**	Line 101, p.6: “We used a qualitative study design (interviews) with practitioners working in child weight management and NCMP delivery.” The authors might want to mention, besides the research design and data-collection strategies, provide the rationale for the design selected.	Thank you for the suggestion, we have now added the following sentence to Line 101: “This method allowed for an in-depth understanding of how the parental guidance was being used in a range of settings and services.”
**3**	Line 116, p.7: “Interviews were conducted online between February-May 2024 by RB, allowing her to develop familiarity and a comprehensive view of the dataset before analysis RB is a female. A point (.) is missing between the two sentences (before RB).	Thank you for spotting this – full stop now added at line 118.
**4**	Line 169, p.10/11: Table 1. Table 1 presents detailed information about the participants; however, it seems somewhat lengthy to include in the body of the article. I leave it to the authors and the journal editor to consider allocating this table to supplementary material, with the presentation of a summary table indicating the absolute/relative frequencies for each response category.	Thank you for this suggestion. We think it is valuable to the reader to have this information about the participants within the body of the paper. However, we have slightly simplified the table (re joint interviews) to make it easier to read.
**5**	Line 332, p.20 “sensitive resource which increased both	Thank you for spotting this – full stop now added at line 324.

	their own and parents' confidence in addressing a sensitive issue The". Please, add a point before the The.	

Supplementary file – COREQ Statement		
	**Reviewer Comment**	**Author Response**
6	Please, change the title according to the last version of the manuscript.	Thank you for spotting this error – title now changed.
7	Use a single way to refer to a page (page or p).	We have now amended this to refer to 'page' throughout the COREQ checklist, for consistency.
8	Section 17. Interview guide. Were questions, prompts, guides provided by the authors? Was it pilot tested?,: It appears question in blue still needs to be answered.	A sentence has now been added into the COREQ, Q17 (before final sentence): "the topic guide was created with input from all authors; it was not pilot tested before use."
